# 3D Printing of Ionogels with Complementary Functionalities Enabled by Self‐Regulating Ink

**DOI:** 10.1002/advs.202302891

**Published:** 2023-06-25

**Authors:** Jiahui Huang, Zhenchuan Yu, Peiyi Wu

**Affiliations:** ^1^ State Key Laboratory of Molecular Engineering of Polymers Department of Macromolecular Science and Laboratory of Advanced Materials Fudan University Shanghai 200433 China; ^2^ State Key Laboratory for Modification of Chemical Fibers and Polymer Materials College of Chemistry and Chemical Engineering Center for Advanced Low‐Dimension Materials Donghua University Shanghai 201620 China

**Keywords:** 3D printing, bio‐inspired materials, direct‐ink‐write (DIW), ionogels, mechanically tunable, soft materials

## Abstract

Shaping soft and conductive materials into sophisticated architectures through 3D printing is driving innovation in myriad applications, such as robotic counterparts that emulate the synergic functions of biological systems. Although recently developed multi‐material 3D printing has enabled on‐demand creation of intricate artificial counterparts from a wide range of functional viscoelastic materials. However, directly achieving complementary functionalities in one ink design remains largely unexplored, given the issues of printability and synergy among ink components. In this study, an easily accessible and self‐regulating tricomponent ionogel‐based ink design to address these challenges is reported. The resultant 3D printed objects, based on the same component but with varying ratios of ink formulations, exhibit distinct yet complementary properties. For example, their Young's modulus can differ by three orders of magnitude, and some structures are rigid while others are ductile and viscous. A theoretical model is also employed for predicting and controlling the printing resolution. By integrating complementary functionalities, one further demonstrates a representative bioinspired prototype of spiderweb, which mimics the sophisticated structure and multiple functions of a natural spiderweb, even working and camouflaging underwater. This ink design strategy greatly extends the material choice and can provide valuable guidance in constructing diverse artificial systems by 3D printing.

## Graphical abstract

This work reports an easily‐accessible and self‐regulating tricomponent ionogel‐based ink design. The resultant 3D printed objects, based on the same component but with varying ratios of ink formulations, exhibit distinct yet complementary properties. The combination of direct ink‐write printing techniques enables the fabrication of bioinspired prototypes of spiderwebs with sophisticated geometry and multiple functionalities.

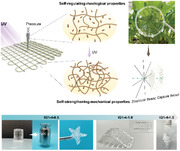

## 1 Introduction

Biological systems provide abundant prototypes that combine mutually complementary functions in a single unit. For instance, spiders spin sticky, extensible, and translucent capture threads on stiff radial structural threads, which synergistically perform the missions of sensing approaching targets, capturing prey, and dissipating the kinetic energy.^[^ ^1^, ^2^, ^3^, ^4^ ^]^ Such integrated features greatly facilitate the advancements in shaping soft conductive materials as robotic counterparts that emulate the synergic functions of biological systems.^[^ ^1^, ^5^, ^6^, ^7^ ^]^ Compared with laborious and cost‐prohibitive mold‐making procedures, emerging 3D printing technologies offer unique advantages in the rapid design and fabrication of objects with complex geometries and functionalities beyond their bulky counterparts.^[^ ^8^, ^9^, ^10^, ^11^, ^12^ ^]^ Among various 3D printing technologies, the extrusion‐based direct ink‐write (DIW) printing technique is of practical importance since it is operationally simple, widely available, and low cost, as long as the precursor ink can be engineered to exhibit proper rheological behavior.^[^ ^13^, ^14^, ^15^ ^]^

Recent advances in DIW‐based 3D printing of soft conductive materials have opened up new opportunities for a broad scope of intelligent applications.^[^ ^10^, ^16^, ^17^, ^18^ ^]^ Hydrogels are known to be a landmark type of soft conductive materials. However, they often suffer from water evaporation and natural non‐conductivity. Instead, ionogels have aroused much attention as an emerging class of soft materials, in which ionic liquids (ILs) are confined in the polymer networks through different internal interactions to form diverse designable structures.^[^ ^19^, ^20^ ^]^ With the combined merits of ILs and polymers including structural diversity, high ionic conductivity, and excellent thermal and chemical stability, ionogels are promising candidates.^[^ ^21^, ^22^ ^]^

In order to satisfy sophisticated properties akin to biological systems, a diverse array of functional viscoelastic materials are often necessary to create intricate 2D or 3D objects, also known as multi‐material 3D printing.^[^ ^10^, ^23^, ^24^ ^]^ Nonetheless, directly achieving complementary functionalities in one ink design remains largely unexplored, as facing the issues of printability and synergy among ink components. For instance, to achieve a printable system, low‐viscosity monomer precursors of ionogels often require the addition of specific rheology modifiers that should be compatible with the ink system, while these rheology modifiers always play a single role in regulating the viscosity. Moreover, directly printing ionogels composed of polymer networks swollen with ILs poses a trade‐off between printability and mechanical strength, because the ease of printing at a relative low polymer content leads to a considerable sacrifice of mechanical robustness, while high strength achieved at a high concentration of polymers makes printing difficult.^[^ ^25^, ^26^, ^27^ ^]^ Given the relatively singular role of each component, it is hard to obtain adjustable performance for their 3D‐printed objects, let alone complementary functions.

In this work, to reconcile the challenges between printability and complementary properties of ionogels, a kind of self‐regulating ink design are adopted. The ink precursors are formulated from three components with structural similarity and excellent compatibility: poly (ionic liquid) networks (PIL), ionic liquid monomers (ILM), and free ionic liquid (FIL) dispersing medium. Adjusting the ratio of their ink precursors allows for precise control of rheological properties and degree of self‐reinforcement with UV post‐treatment, thus enabling efficient DIW printing and tailored mechanical properties of printed structures. The Young's modulus of the printed structures can differ by three orders of magnitudes (from 521.3 kPa to 148.7 MPa), featuring either rigidity or stretchability and adhesiveness. On this basis, we further demonstrate a representative bioinspired prototype of spiderweb by 3D printing mechanically complementary ionogels with sophisticated geometry (e.g., stiff structural threads and sticky and stretchable spiral threads) and multiple functionalities (e.g., capturing, sensing, and dissipating energy). The printed synthetic spiderweb is amphibious and has been shown to synergistically execute the missions of capturing and sensing in both ambient and aquatic environments. The integration of design freedom and available functionalities from diversified ionogels not only broadens the design of artificial systems but also extends their scope of applications.

## 2 Results and Discussion

### 2.1 Ink Design with Self‐Regulating Rheological Properties

**Figure 1:**
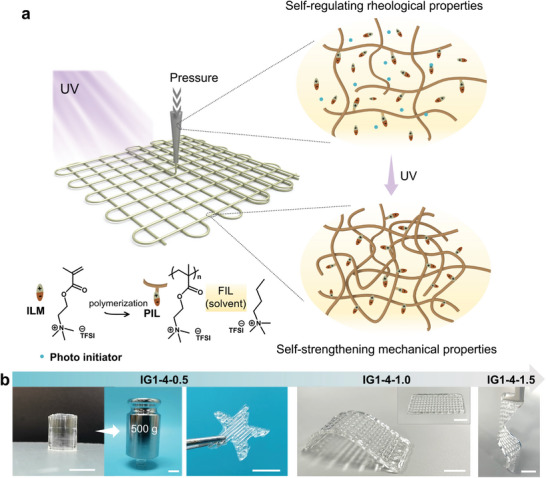
Schematic design of the 3D printing process. a) Schematic diagram of the 3D printing process and corresponding structural changes. b) Digital photos of printed patterns and scaffolds with tunable mechanical properties. (Scale bars: 1 cm).

The developed 3D printing procedure involves DIW printing of precursor inks and post‐print polymerization, as schematically represented in **Figure** **1**a. Rational design of self‐thickening ink formulations is key in our 3D printing strategy, which is composed of three main components: poly (2‐(methacryloyloxy) ethyl trimethylammonium bis(trifluoromethanesulfonyl)imide) (poly[EMTMA][TFSI]) as poly (ionic liquid) networks (PIL), [EMTMA][TFSI] as uncured ionic liquid monomers (ILM), and butyltrimethylammonium bis(trifluoromethanesulfonyl)imide ([N_4111_][TFSI]) as free ionic liquid (FIL) dispersing medium. The three components were synthesized by the facile anion exchange method, with bis (trifluoromethylsulfonyl)imide (TFSI^−^) as the counter‐anion (Figure S1, Supporting Information).^[^ ^28^ ^]^ In this case, their ammonium cationic units exhibit structural similarity and share the same anion, ensuring strong affinity and excellent compatibility.

Thus, homogeneous PIL‐ILM‐FIL ink can be obtained upon mixing and is named Ink a‐b‐c, where numbers a, b, and c represent the ratio of the weights of PIL, ILM, and FIL, respectively (a series of ink formulations are given in Table S1, Supporting Information). The as‐prepared ink is extrudable and can be printed into geometrically complex architectures through the layer‐by‐layer deposition (Figure S2 and Movie S1, Supporting Information). Each of these three components plays an important role in the precursor ink. Specifically, PILs are polymers whose backbones consist of IL monomers (repeat units),^[^ ^29^ ^]^ which can not only effectively bind and stabilize ionic species within polymer networks, but also function as a rheological modifier to increase the viscosity of the ink. ILM and FIL can fully swell the polymer networks, and meanwhile work together to modulate the rheological properties (i.e., yield stress and viscosity) of the ink due to the plasticizing effect of ionic liquid.^[^ ^30^ ^]^

In the subsequent post‐printing treatment step, the printed constructs are exposed to UV light to initiate the polymerization of ILMs in the presence of a small amount of photoinitiator for enhancing the long‐term shape stability. During this process, ILMs transform from solvent into a percolating self‐strengthening network and only FIL remains as dispersion medium, allowing the regulation of the mechanical properties of the printed structures. The samples undergoing post‐printing treatment are noted as IG a‐b‐c correspondingly. By precisely tuning the ratio of the ternary ink, the printed 3D structures present customizable mechanical properties, as shown in Figure 1b. For example, the printed cylindrical geometry of IG 1‐4‐0.5 is rigid to sustain a load of 500 g, ≈ 500 times its own weight. While the printed grid‐structured film of IG 1‐4‐1.5 is flexible and stretchable. Due to the excellent compatibility and the established ion‐dipole and ion–ion interactions between PIL, ILM, and IL, the resultant IGs are highly transparent without phase separation and almost visually imperceptible in the ambient environment. The average transmittance is > 94% at a thickness of 1 mm in the visible region (400–700 nm, Figure S3, Supporting Information). We believe the design principles of this ternary system shed light on the preparation of a variety of printable inks with tunable rheological and mechanical properties.

**Figure 2:**
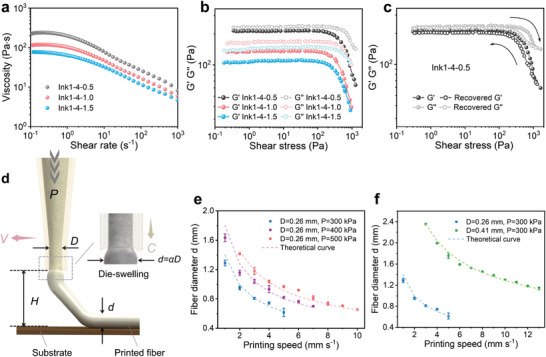
Self‐regulating rheological properties of designed ink formulations. a) Shear‐thinning behavior of the three inks. b) Log–log plot of storage modulus (G′) and loss modulus (G″) as a function of shear stress for three ink formulations. c) Recovery measurement of Ink1‐4‐0.5, which sweeps from low stress to high stress and back to low stress. d) Schematic illustration of the typical printing settings of DIW 3D printing. Comparison of experimental data with theoretical data of Ink1‐4‐0.5 at e) different air pressure P and f) different nozzle diameter D. Data in (e,f) are means ± S.D., *n* = 3.

Owing to the high density of polar C–F groups and Coulomb potential between cation and anion units, ion–dipole and ion–ion interactions can be established in the precursor ink.^[^ ^25^, ^27^, ^31^, ^32^ ^]^ A series of rheological measurements were then carried out to probe the viscoelastic properties and printability of inks. As plotted in **Figure** **2**a, the formulated Ink1‐4‐0.5, Ink1‐4‐1.0, and Ink1‐4‐1.5 with the same components but different ratios all display desired shear‐thinning behavior, as evidenced by the decrease in apparent viscosity (*η*) by about two orders of magnitude with increasing applied shear rates ($\dot{\gamma }$). The lower viscosity under high shear rates guarantees the ease of extrusion through fine nozzles, while the high viscosity at low shear rates contributes to preserving the printed shape.^[^ ^33^, ^34^ ^]^ This behavior can be quantified via the power‐law viscosity model, $\eta =K\;{\dot{\gamma }}^{n-1}$, where *K* is the consistency index and *n* is the flow index. The flow index *n* describes the rate dependence of the fluid; that is, for shear‐thinning fluids, 0<*n*<1, while for shear‐thickening, *n*>1.^[^ ^13^, ^35^, ^36^ ^]^ By fitting in the shear rate region of 1 to 1000 s^−1^, the flow index *n* is calculated to be 0.54, 0.58, and 0.61 for Ink1‐4‐0.5, Ink1‐4‐1.0, and Ink1‐4‐1.5, respectively, consistent with the properties of shear‐thinning fluids. In the oscillatory stress sweep testing, the three inks demonstrate semi‐solid‐like behavior at the wide plateau regions (G′ is independent of stress), where the dynamic storage modulus (G′) is very close to the loss modulus (G″), indicating the formation of a viscoelastic network by the entanglement of PIL macromolecular chains. The increased shear stress makes G″ values much greater than G′, and results in the appearance of yield stress (the stress that has to be exceeded for deformation to occur).^[^ ^36^, ^37^ ^]^ As the content of PIL increases, the value of yield stress increases as well, reaching 344, 350, and 454 Pa for Ink1‐4‐1.5, Ink1‐4‐1.0, and Ink1‐4‐0.5, respectively, which confirms the thickening effect from flexible macromolecular backbones (Figure 2b; Figure S4, Supporting Information). Moreover, cyclic oscillatory measurements from low to high and back to low shear stress reveal the reversible changes in modulus (Figure 2c; Figure S5, Supporting Information). This allows the ink to be extruded at relatively low pressure and maintain its shape fidelity once discharged from the printing head.

As a viscoelastic ink suitable for DIW‐based printing, the cross‐sectional geometry of the printed filaments is almost identical in different cases, thus making it possible to predict and control the printing resolution.^[^ ^38^ ^]^ Figure 2d schematically illustrates the typical settings and parameters for DIW 3D printing. Generally, under the applied air pressure *P*, the ink is extruded at a speed of *C* from the nozzle tip with the inner diameter *D*. The extrusion of viscoelastic ink would induce the die‐swelling of the extruded filament, making the extruded filament diameter *αD* different from the nozzle diameter *D*.^[^ ^39^ ^]^ *α* is known as the die‐swelling ratio and often related to polymer material properties, die diameter, air pressure, etc.^[^ ^38^, ^40^ ^]^ Meanwhile, the nozzle moves at a speed of *V* to deposit the printed filaments, during which the straight fibers will be towed and stretched, resulting in the diameter of printed filament *d* being smaller than that of extruded filament *αD*. Previous studies have shown that the effect of air pressure *P* and nozzle diameter *D* on filament diameter *d* follows $d=\alpha D/\sqrt{V/C}$.^[^ ^40^ ^]^ Taking Ink1‐4‐1.5 as an example, as de in Figure 2e, at the fixed printing speed *V*, the filament diameter *d* is larger at a higher air pressure *P* (i.e., 500 kPa). This is because increasing air pressure *P* would increase both the die‐swelling ratio *α* and extrusion speed *C*. In Figure 2f, increasing the nozzle diameter *D* leads to an increase in extrusion speed *C*, while the die‐swelling ratio *α* decreases. Under the combined effect of *D*, *C*, and *α*, in the same printing speed and pressure, the filament diameter *d* increases with the increased nozzle diameter *D*. More detailed analysis can be seen in Note S1 (Supporting Information). By choosing the appropriate printing parameters, the sub‐millimeter resolution of ≈650 µm can be achieved.

For the purpose of predicting and controlling the diameter, according to the volume conservation, the above relation can be expressed as $d_{2}=d_{1}\sqrt{V_{1}/V_{2}}$, where *d_1_* is the surveyed filament diameter and *d_2_* is the intended filament diameter. *V_1_* and *V_2_* refer to the printing speed before and after the speed variation, respectively. Based on this theoretical relation, measuring the primary diameter *d_1_* at one arbitrary speed *V_1_* in advance, the diameter of printed filament at different printing speeds can be easily predicted through a facile calibration procedure. As plotted in Figure 2e,f, the printing speed *V_1_* of 3 mm s^−1^ is chosen, and the theoretical curves show great accuracy with experimental data under different air pressure and nozzle diameter, which means this model can effectively guide rational selection of printing parameters in our ink system.

### 2.2 Self‐Strengthening and Tailored Complementary Properties

**Figure 3:**
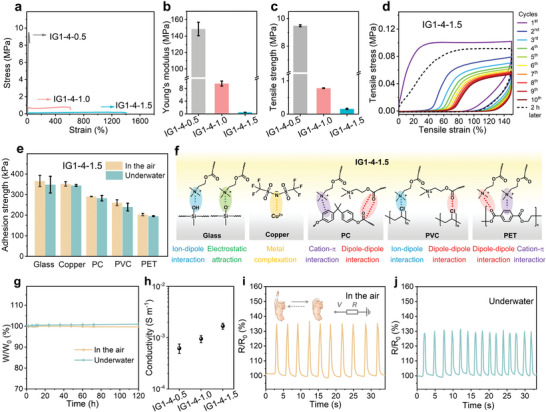
Self‐strengthening and tailored mechanical properties. a) Stress–strain curves, b) Young's modulus, and c) tensile stress of IG1‐4‐0.5, IG1‐4‐1.0, and IG1‐4‐1.5, respectively. d) Cyclic stress–strain curves of the IG1‐4‐1.5. e) Adhesion strength of IG1‐4‐1.5 for different substrates in the air and underwater. f) The possible adhesion mechanisms between the ionogel and various substrates. g) Weight change of the IG1‐4‐1.5 as a function of time. *W_0_* and *W* correspond to the initial weight, and the weight at different times, respectively. h) Ionic conductivity of ionogels with different contents of IL. Resistance changes of artificial finger bending and releasing movements monitored by IG1‐4‐1.5 based sensor i) in the air and j) underwater. Data in (b,c,e,h) are means ± S.D., *n* = 3.

Apart from the self‐regulating rheological properties of precursor inks, the post‐printing treatment initiates the polymerization of ILMs and facilitates the formation of a self‐strengthening network, thus endowing the printed objects with impressive and adjustable mechanical properties. The disappearance of the C=C stretching vibration band of IGs (1639 cm^−1^) in Fourier transform infrared (FTIR) spectra verifies the successful polymerization of the secondary self‐reinforced network (Figure S6, Supporting Information). At the same time, the asymmetric bending of CF_3_ (1175 cm^−1^) in IG slightly shits to lower wavenumber, indicating the ion–dipole interaction between cation from FIL and the CF_3_ group of PIL. The mechanical properties were then quantified through tensile tests. As shown in **Figure** **3**a–c, the stretchability, modulus and tensile strength of the three post‐cured samples significantly vary in a wide range. As a result of the strong internal ionic interactions and the entanglement of polymer backbones, the IG1‐4‐0.5 is stiff and displays the highest Young's modulus of 148.7 MPa and tensile stress of 9.4 MPa. As increasing the content of FIL, the IG1‐4‐1.5 show a higher strain (≈1400%) but lower Young's modulus (521.3 kPa) and tensile stress (12.1 kPa). The distinct mechanical differences can be explained by the plasticizing effect of free ionic liquids and thus the enhanced chain flexibility.^[^ ^30^, ^41^ ^]^ Notably, the Young's modulus of IG1‐4‐0.5 shows three orders of magnitude larger than that of IG1‐4‐1.5, which is similar to spiderwebs, as the elastic modulus of structural threads and capture threads can differ by three orders of magnitudes.^[^ ^3^, ^42^ ^]^

More importantly, the resulting IG1‐4‐1.5 affords effective energy dissipation capability, outstanding adhesion, stability and sensing ability, which is coincide well with multi‐functions of the capture threads of a spiderweb, such as capturing, buffering, sensing, etc. Cycling tensile curves in Figure 3d demonstrate the energy dissipation ability of IG1‐4‐1.5, as evidenced by the large hysteresis for the first loading–unloading cycle, yet basically recovers to the original loading curve after a relatively short waiting time (≈120 min). This suggests that the multiple dynamic interactions in the ionogel could reversibly break and reform.^[^ ^43^ ^]^ The as‐prepared IG1‐4‐1.5 also achieves favorable compliant adhesion performance to a wide variety of substrates in the air, which was evaluated by the lap shear test. The adhesion strength of IG1‐4‐1.5 to glass, copper, polycarbonate (PC), polyvinyl chloride (PVC), and polyethylene terephthalate (PET) is plotted in Figure 3e. Among the investigated substrates, the IG1‐4‐1.5 shows the maximum adhesion strength of 365.6 ± 27.3 kPa on the glass substrate, outperforming many catechol‐based adhesive hydrogels.^[^ ^44^, ^45^ ^]^ Such versatile adhesion behavior can be attributed to abundant functional groups (i.e., ammonium cations and carbonyl groups) in the ionogel, which can produce selective and robust bonding with diverse substrates via ion‐dipole interactions, electrostatic interactions, dipole–dipole interactions, metal complexation, and cation–*π* interactions, as sketched in Figure 3f.^[^ ^25^, ^27^, ^46^, ^47^, ^48^, ^49^ ^]^ Especially, most hydrogels and ionogels still struggle to achieve robust adhesion underwater. Because the interfacial water could separate the molecules of the two surfaces and consequently prevents the interfacial adhesion in the aquatic environment.^[^ ^50^ ^]^ Thanks to the fact that C—F bond is a very poor hydrogen bonding acceptor,^[^ ^31^, ^51^ ^]^ the presence of hydrophobic anion (TFSI^−^) in our ionogel would expel or destroy the hydration layer on the surface, endowing IG1‐4‐1.5 with strong underwater adhesion comparable to that in the air (Figure 3e). The fluorine‐rich matrix also guarantees high stability and tolerance against water, suggesting their potential underwater uses. As shown in Figure 3g, the weight of our ionogel shows negligible drift when stored at the ambient condition and underwater for 10 days, respectively. The remarkable stability is further affirmed by differential scanning calorimetry (DSC) measurements (Figure S7, Supporting Information). Under a wide range of temperatures (−45 to 100 ^o^C), there is no melting point or glass transition observed in our ionogel.

As well as tunable mechanical properties, the ionogels demonstrate good conductivity and sensing capabilities derived from their ionic polymer skeleton and free ionic liquids. As shown in Figure 3h, their conductivity can be varied from 6.2×10^−4^ to 1.7×10^−3^ S m^−1^ by increasing the concentrations of FIL, since the ionic conductivity is usually proportional to the effective number of mobile ions. As a result, one piece of IG1‐4‐1.5 can serve as the sensing layer and be easily assembled into an ionic resistor after wiring (Figure 3i). The fabricated IG1‐4‐1.5‐based sensor delivers repeatable and strain‐dependent electrical responses when attached to a prosthetic finger, and works even underwater (Figure 3j). Also, it performs excellent segment stability when loaded with the step‐up strains, and then unloaded to the initial state without obvious resistance changes (Figure S8, Supporting Information). Such remarkable sensing properties and broad working environments readily support their utilization for practical applications.

### 2.3 3D‐Printed Synthetic Spiderwebs based on Ionogels with Complementary Functions

**Figure 4:**
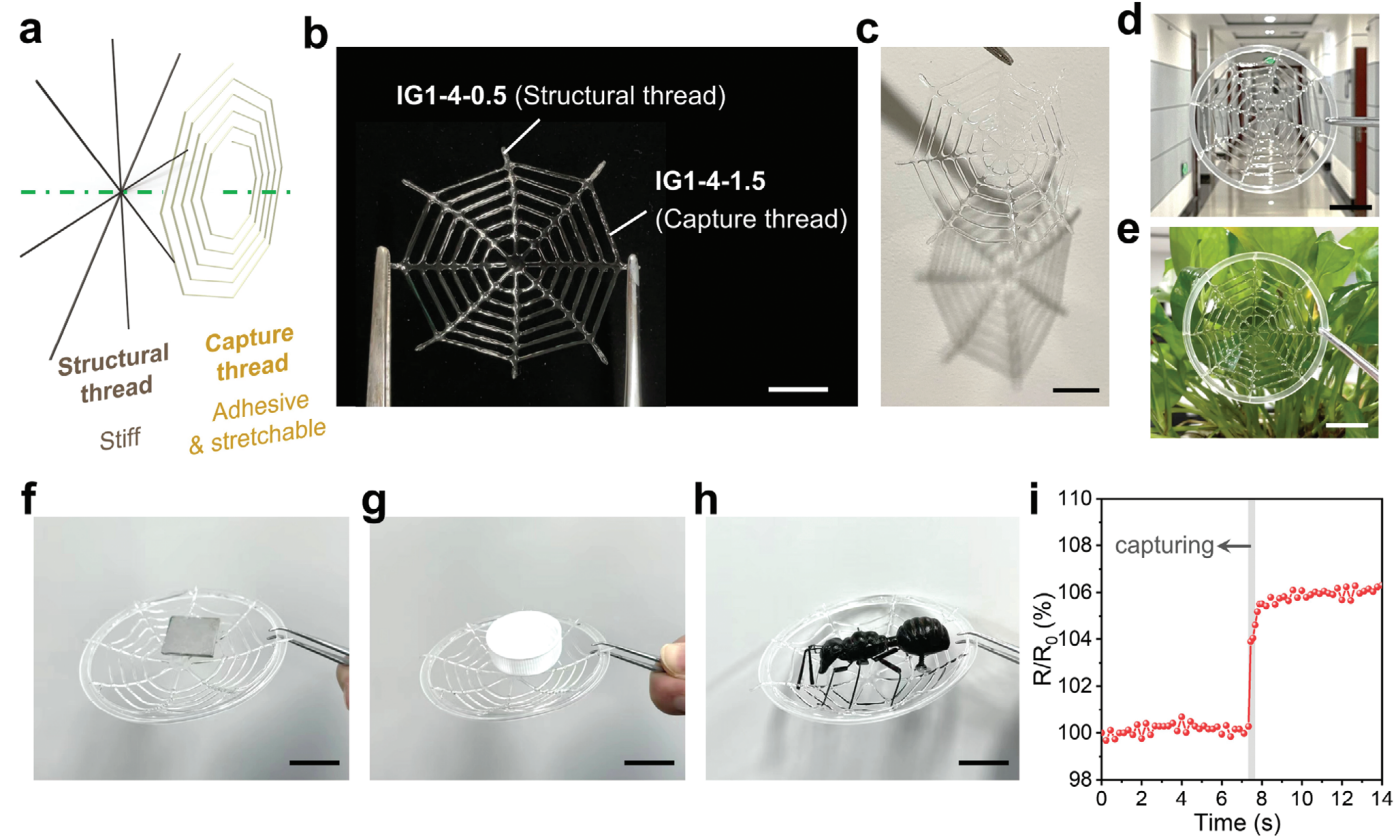
3D‐printed synthetic spiderwebs that can work in the air. a) Schematic of the exploded view of orb spiderwebs. Spiders can spin stretchable, adhesive, and translucent capture threads on strong structural threads. b) A 3D‐printed spiderweb based on ionogels with distinct mechanical properties to mimic different parts of the spiderwebs. c–e) The printed spiderweb is transparent and can be passively camouflaged in a variety of environments. Capturing performance of printed spiderwebs on various objects, including f) a piece of iron (1 g), g) a bottle cap (1.7 g), and h) a plastic insect (7.5 g). i) Corresponding electrical signals when capturing a plastic insect from (h). Scale bars: 2 cm.

Different ink formulations based on same components result in distinct but complementary properties, which encourage us to print spiderweb geometry to emulate the complementary properties and functions of natural orb webs. The stiff IG1‐4‐0.5 can be selected as the radial threads to support the overall structure of the web, and the adhesive and stretchable IG1‐4‐1.5 can be printed as the capture threads for catching and energy dissipation (**Figure** **4**a,b).

Figure 4c shows a 3D‐printed integrated web structure with perfectly connected junctions, and the strong interlayer adhesion ensures the web integrity. In diverse environments such as corridors and in front of plants (Figure 4d,e), the printed synthetic spiderweb displays passive camouflage thanks to the high transparency of ionogels. Here the 3D printing technique provides a pathway to introduce design freedom, complementary functions, and stability into the printed structure, which is difficult to achieve via mold casting. Therefore, under the impact from external objects (e.g., a piece of iron, a bottle cap, and a plastic insect), the as‐fabricated spiderweb remains intact and is able to catch these items firmly like a real spiderweb does (Figure 4f–h). Besides, this capture process can be recorded with electrical signals based on the reliable ion conduction mechanism. As depicted in Figure 4i, when the plastic insect falls on the synthetic spiderweb, its gravity causes the deformation of the extensible capture threads, thus resulting in a transient rise in resistance. Notably, capturing and sensing take place simultaneously, without taking time to switch the two modes.

**Figure 5:**
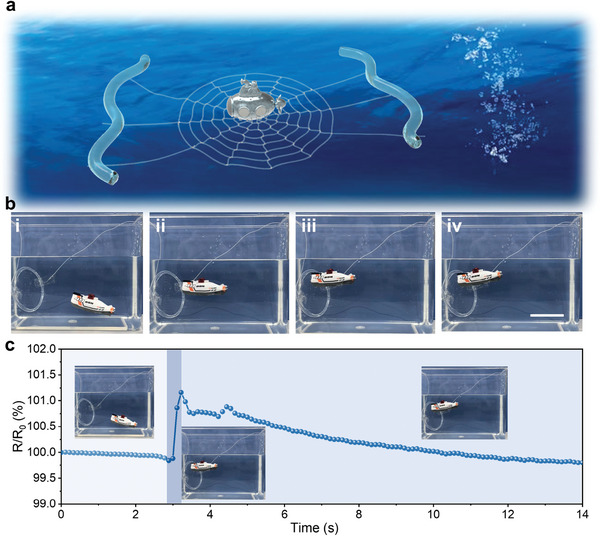
3D‐printed synthetic spiderwebs that can work and camouflage in the aquatic environment. a) Schematic diagram of underwater capture and sensing using the printed synthetic spiderweb. b) A submarine model is shown approaching the printed synthetic spiderweb underwater and then being securely captured by it. The printed spiderweb is highly transparent, allowing optical camouflage in the aquatic environment. Scale bar: 5 cm. c) Corresponding electrical response in the process of underwater capturing. Inset photos are captured during measurements from Movie S2 (Supporting Information).

In recent decades, there has been an increasing interest in the exploration and exploitation of the oceans due to their enormous resources and special military strategic position. The development of underwater spiderwebs for marine survey and foreign object capture is meaningful yet challenging for marine exploration and the military.^[^ ^25^ ^]^ The excellent stability of the ionogels underwater also makes it possible for synthetic spiderwebs to work in the aquatic environment (**Figure** **5**a).

As a proof‐of‐concept, we present a demo of synchronous capture and detection of a submarine model using our synthetic spiderweb (Figure 5b and Movie S2, Supporting Information). The printed spiderweb is first fixed in water, with its structural threads anchored to a circular PE framework and its capture threads connected with metal electrodes (Figure 5b‐i). Its high transparency allows it to be optically camouflaged even in aquatic environments. Subsequently, the electric submarine weighing 20 g sails toward the synthetic spiderweb under remote control at a speed of ≈ 0.05 m s^−1^ (Figure 5b‐ii; Figure S9, Supporting Information). The control over the electric submarine is stopped when it contacts the spiderweb (Figure 5b‐iii). Due to the adhesion and buffering synergy of the capture threads, the submarine can be firmly captured by the spiderweb (Figure 5b‐iv). Along with effective capture, the targets can be tracked and sensed in the form of resistive signals, and the corresponding electrical response is shown in Figure 5c. When the submarine hits the spiderweb, the real‐time resistive signals increase immediately and recover in several seconds, probably due to the buoyancy in the water. Therefore, the surge in resistance signals can indicate that the approaching targets come into contact with the synthetic spiderweb, which also helps prevent the target from escaping. This demonstrates the great potential of our printed synthetic spiderweb to synergistically complete complex missions in multiple environments yet fabricated in a facile way.

## 3 Conclusion

We here propose an easily‐accessible and self‐regulating tricomponent ionogel‐based ink design to produce 3D architectures with complementary properties. First, the rational choice of self‐thickening ternary ink formulations composed of PIL, ILM, and FIL is the key to our 3D printing strategy. By adjusting the ratio of their ink precursors, precise control of rheological properties and degree of self‐reinforcement can be achieved, which enables efficient printing and customized distinct mechanical properties of printed structures. A theoretical model is also employed for choosing appropriate printing parameters, making it possible to predict and control the printing resolution.

Second, to mimic the real spiderwebs system, the stiff IG1‐4‐0.5 with the highest Young's modulus (148.7 MPa) and tensile stress (9.4 MPa) is chosen as the structural threads, and the adhesive and translucent IG1‐4‐1.5 with the highest strain (≈1400%) but lower Young's modulus (521.3 kPa) is selected as the capture threads, as the elastic modulus of structural threads and capture threads can differ by three orders of magnitudes. This design makes the synthetic spiderweb realize sensing and capturing, as a natural spiderweb does. With the combined advantages of ionogels in materials and 3D printing technique in processing, the synthetic spiderweb functions in the aquatic environment as well, with the extended scope of working environments. In a proof‐of‐concept demonstration, this synthetic spiderweb can camouflage underwater and meanwhile capture and sense approaching targets synchronously. The easy accessibility of this ink design strategy greatly bridges the design challenge and the application realization for creating artificial systems with sophistication in architecture and performance.

## 4 Experimental Section

### Materials

Bis(trifluoromethanesulfon)imide lithium salt (LiTFSI) and 2,2‐diethoxyacetophenone (photoinitiator) were purchased from Aladdin Co. 2‐(Methacryloyloxy)ethyl trimethylammonium chloride ([EMTMA][Cl]) (75 wt% in water) and 2,2‐azobis(2‐ methylpropionamidine) dihydrochloride (AIBN) were purchased from Sigma–Aldrich. Butyltrimethylammonium chloride ([N_4111_][Cl]) was got from Energy Chemical. Acetone was purchased from Sinopharm Chemical Reagent Co. All reagents were used without further purification. The weight of the piece of iron, bottle cap, and a plastic insect in Figure 4f–h were 1, 1.7, and 7.5 g, respectively. The weight of the submarine model was 20 g.

### Preparation of Polymerizable Ionic Liquid Monomer (ILM)

The method of preparing polymeric ionic liquids was referred to literature,^[^ ^28^ ^]^ as shown in Figure S1a (Supporting Information). In brief, 1 m [EMTMA][Cl] solution and 1 m LiTFSI were mixed and stirred for 2 h. After the phase separation occurred, the oil phase at the bottom was collected and washed with deionized water three times. Transparent liquid of [EMTMA][TFSI] was finally obtained by vacuum drying at 70 ^o^C for 12 h.

### Synthesis of Poly (Ionic Liquid) (PIL)

The method of preparing polymeric ionic liquids was referred to literature,^[^ ^28^ ^]^ as shown in Figure S1b (Supporting Information). Briefly, [EMTMA][Cl] (30 g) and thermal initiator AIBN (0.12 g) was dissolved in distilled water (300 mL) one after another. Then the free radical polymerization was carried out at 70 ^o^C for 6 h under nitrogen atmosphere. Afterward, the resulting poly([EMTMA][Cl]) solution was dialyzed for five days and freeze‐dried. To prepare poly([EMTMA][TFSI]), a simple anion exchange method was used. poly([EMTMA][Cl]) (10 g, 0.0483 mol) was dissolved in distilled water (150 mL) and then mixed by stirring with an excess of 1 m LiTFSI solution (50 mL). Due to the anion exchange reaction, the Cl^−^ in poly([EMTMA][Cl]) was exchanged by TFSI^−^ and precipitated in the aqueous media. After being washed with an excess amount of water, filtered, and freeze‐dried, polymeric ionic liquid poly([EMTMA][TFSI]) was attained.

### Synthesis of Free Ionic Liquid (FIL)

Free ionic liquid [N_4111_][TFSI] was prepared in the same way as [EMTMA][TFSI], except that [EMTMA][Cl]was replaced by [N_4111_][Cl], as shown in Figure S1c (Supporting Information).

### Preparation of Ionogel Inks

Inks were prepared by dissolving PIL (poly([EMTMA][TFSI])) in the ILM ([EMTMA][TFSI]) and solvent [N_4111_][TFSI] with the help of acetone. After homogeneous mixing, acetone was removed by vacuum drying at 50 ^o^C for 12 h. The finally obtained inks were named Ink a‐b‐c, where numbers a, b, and c represented the ratio of the weights of PIL, ILM, and FIL, respectively. Take Ink1‐4‐0.5 as an example, PIL (1 g) was dissolved in ILM (4 g), FIL (0.5 g), and acetone (1 mL) with stirring overnight, then dried at 50 ^o^C for 12 h to remove the acetone.

### 3D Printing Procedure

All printings were carried out by a precision 3D printing system (3D Bio‐Architects working station, Regenovo). Various configurations of 3D printed sheets were designed with commercial software (3ds Max) and converted to G‐codes that determine the printing pathways. Syringes, tapered dispensing needles with different inner diameters of the needle tip (0.26–0.41 mm), and digital pneumatic regulator were configured for extrusion. The viscosity of the ionogel ink was decreased due to the shear‐thinning effect and then recovered to high viscosity upon exiting. A UV LED (365 nm, 20 W) was used to initiate the curing of the ink as it was extruded from the syringe. The distance of UV light source from the printed samples was ≈ 20 cm. The printing process was carried out at 25 ^o^C. The specific printing parameters for each ink were shown in Table S1 (Supporting Information). The sample was referred to as IG a‐b‐c, where numbers a, b, and c represented the ratio of the weights of PIL, ILM, and FIL, respectively.

### Characterizations

*Rheological characterization*: The rheological properties of inks were measured by a HAAKE MARS modular advanced rheometer equipped with 25 mm parallel plate geometry. Viscometry measurements were conducted over shear rates from 0.1 to 1000 s^−1^. Oscillatory measurements were carried out at a frequency of 1 Hz. The tests were all performed at 25 °C.

### Optical Characterization

The transmittance of the ionogels was measured with a Lamda 35 UV–vis spectrophotometer (Shimadzu, Japan). Fourier transformed infrared (FTIR) spectra was recorded on a Thermo Fisher spectrometer (Nicolet 6700, USA) using attenuated total reflectance (ATR) method. The fiber diameters under different printing parameters were taken by an optical microscope (Leica DM2500P).

### Mechanical Test

The tensile test was carried out on a universal testing machine (Instron 5966, USA) at a stretch rate of 100 mm min^−1^. For the cyclic tensile test, both loading and unloading were performed at a constant velocity of 100 mm min^−1^.

### Adhesion Measurement

The adhesion strength was measured using the typical lap shear test. Before the tests, all the substrates were washed sequentially with acetone, DI water, and ethanol, followed by complete drying at the ambient. A small piece of IG1‐4‐1.5 was sandwiched between several substrates with a joint area of 1.5 cm^2^. The adherent joint was then pressed with a preload for 12 h as a sample to test the adhesion strength in the air. As the samples to test the adhesion strength underwater, the above steps were performed underwater. After this, the samples were stretched on both ends by tensile machine (Instron 5966, USA) at the speed of 50 mm min^−1^. The maximum tensile force at joint failure divided by the overlap area was calculated to be the adhesion strength. At least three samples were examined and averaged data were reported.

### Stability Assessment

Differential scanning calorimetry (DSC) curves were recorded with a TA Q800 instrument at a scanning rate of 10 K min^−1^ in a nitrogen flow.

### Electrical Characterization

Ionic conductivity measurement was carried out on a CHI660E electrochemical workstation using AC impedance spectroscopy between 0.1 MHz and 1 Hz. The real‐time resistance changes were recorded by an LCR meter (TH2830) at the alternating‐current (AC) voltage of 1 V and the sweeping frequency of 1 kHz.

### Statistical Analysis

All the results in this study were presented as mean ± S.D., and all the mechanical and electrical properties presented in this study were measured from at least three parallel samples. The statistical analyses were carried out with the OriginPro 2021 software.

## Conflict of Interest

The authors declare no conflict of interest.

## Supporting information

Supporting Information

Supplemental Movie 1

Supplemental Movie 2

## Data Availability

The data that support the findings of this study are available from the corresponding author upon reasonable request
