# Supplementary material for: 3D Printing of Ionogels with Complementary Functionalities Enabled by Self‐Regulating Ink
Source: Adv Sci (Weinh). 2023 Jun 25;10(24):2302891. doi: 10.1002/advs.202302891 (PMC10460849; doi:10.1002/advs.202302891)
Supplement: Supplementary file 1 — Supporting Information [file ADVS-10-2302891-s002.pdf]

ADVANCED SCIENCE
Open Access

Supporting Information

for *Adv. Sci.*, DOI 10.1002/advs.202302891

3D Printing of Ionogels with Complementary Functionalities Enabled by Self-Regulating Ink

*Jiahui Huang, Zhenchuan Yu and Peiyi Wu**

Supporting Information

3D Printing of Ionogels with Complementary Functionalities Enabled by Self-regulating Ink

*Jiahui Huang, Zhenchuan Yu, and Peiyi Wu**

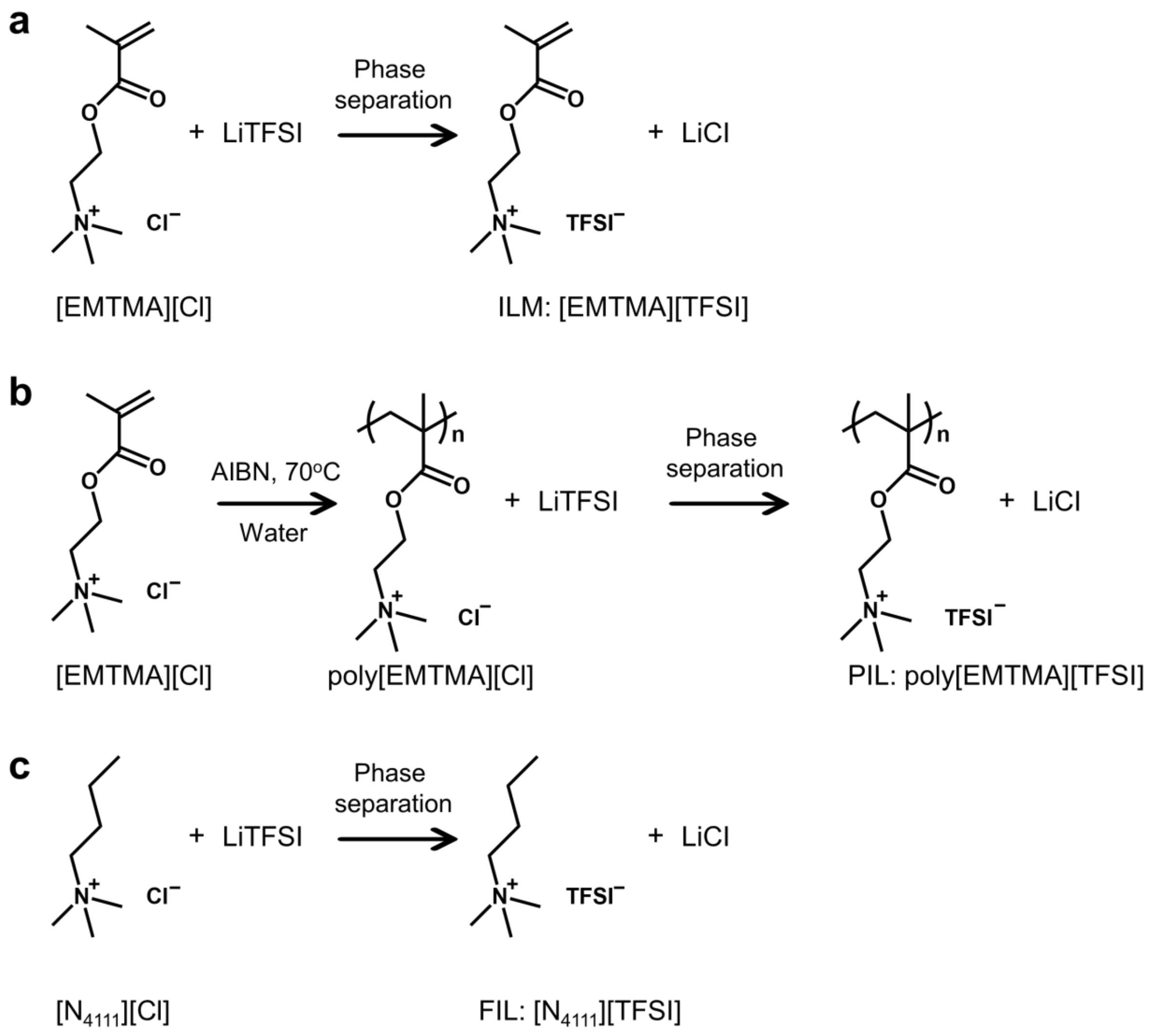

Figure S1. Schemes illustrating the synthesis of (a) ionic liquid monomers (ILM), (b) pre-crosslinked ionic liquids (PIL), and (c) free ionic liquid solvent (FIL).

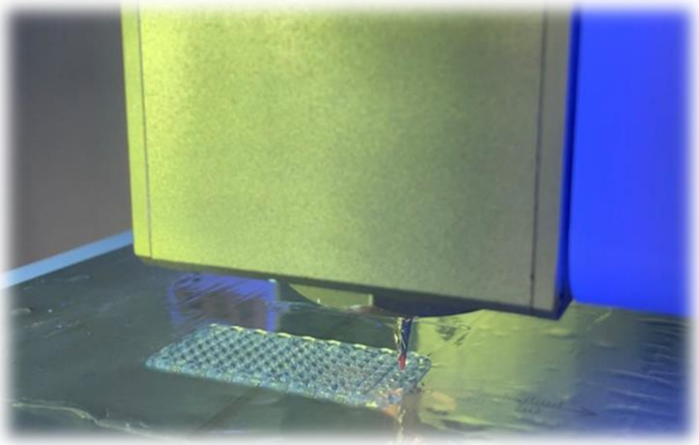

Figure S2. Photograph of 3D printing process with the UV light on.

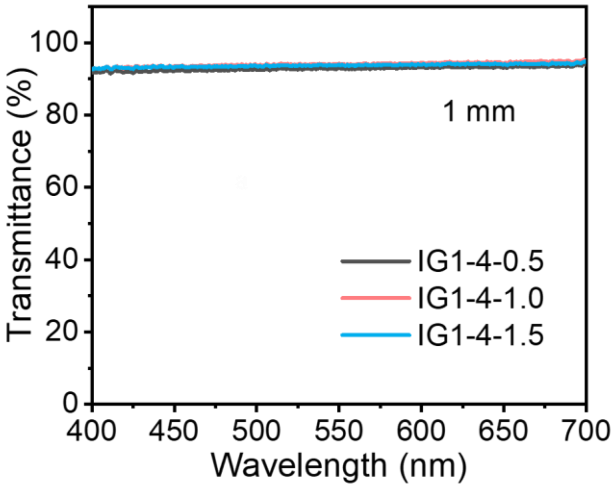

Figure S3. The transmittance of ionogels with thicknesses of 1 mm in the visible wavelength range of 400-700 nm.

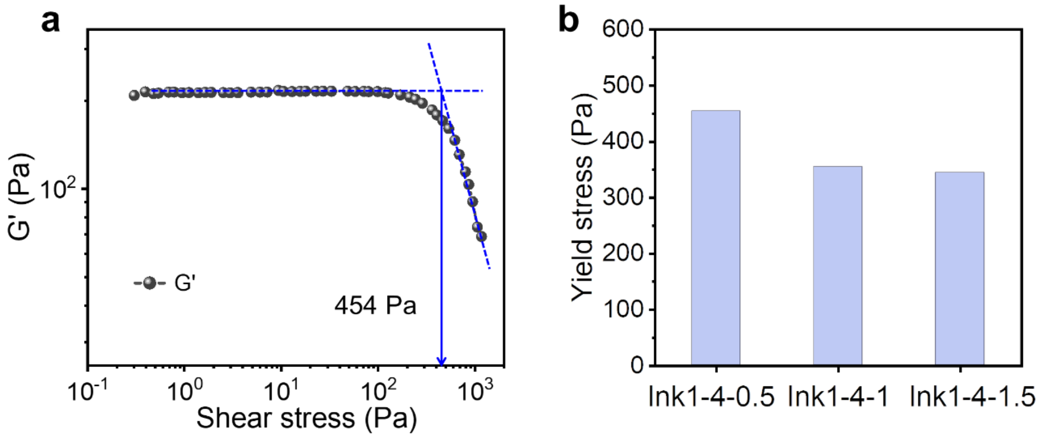

Figure S4. (a) Log-log plot of shear storage modulus (G′) as a function of shear stress for Ink1-4-0.5 and the method to determine the yield stress. The yield stress is taken as the onset value of the downward turn of the modulus curve. (b) Calculated yield stress for Ink1-4-0.5, Ink1-4-1, and Ink1-4-1.5 form Figure 3b.

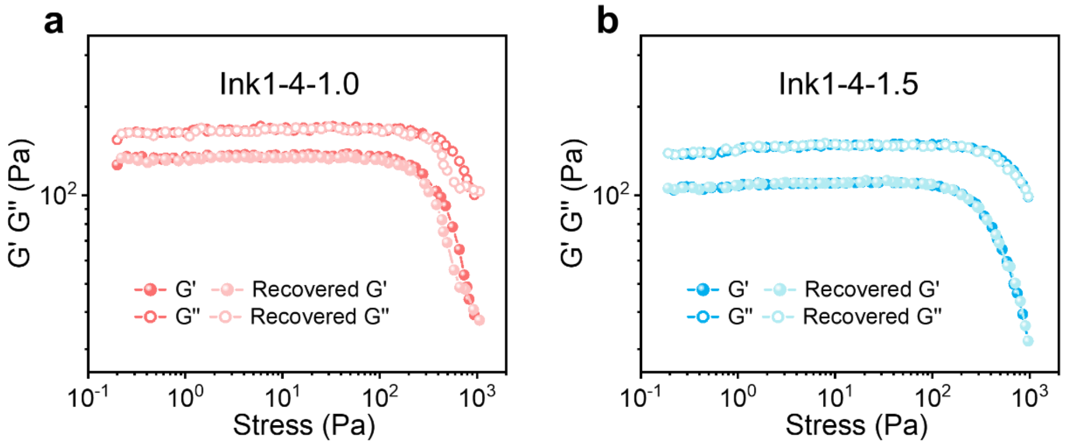

Figure S5. Recovery measurements of (a) Ink1-4-1.0 and (b) Ink1-4-1.5, which sweep from low stress to high stress and back to low stress.

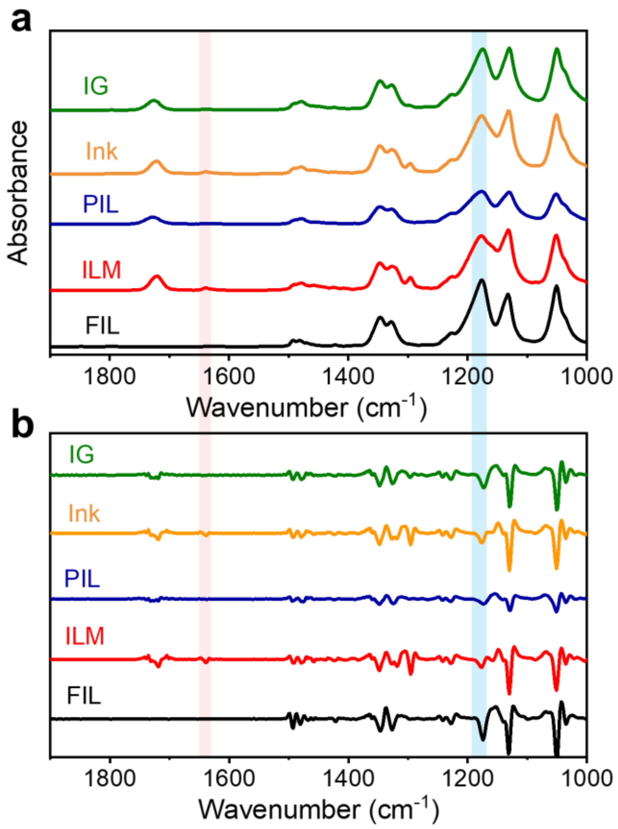

Figure S6. (a) FTIR spectra and (b) the corresponding second derivative curves of FIL, ILM, PIL, Ink, and IG, respectively.

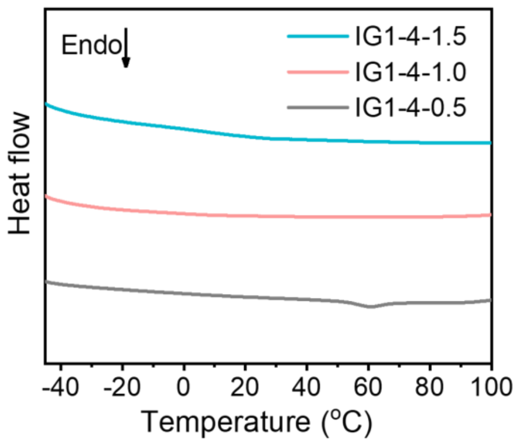

Figure S7. DSC profiles of IG1-4-0.5, IG1-4-1.0, and IG1-4-15, respectively.

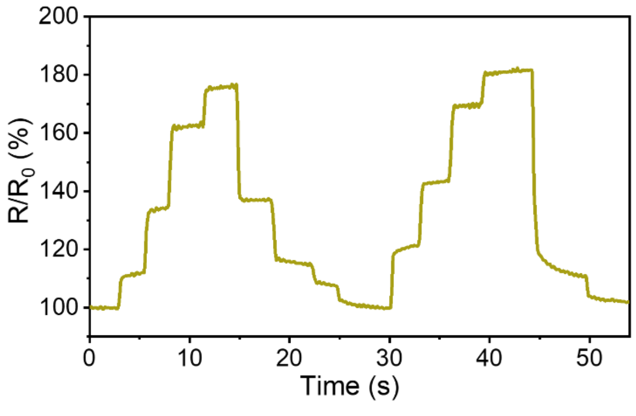

Figure S8. Resistance response of the IG-1-4-1.5 based sensor under a series of step-up strains and back to the initial state, which is tested in the air.

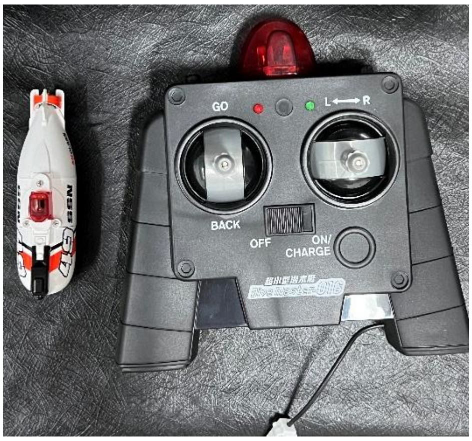

Figure S9. Digital photo of submarine model and its remote control.

Note S1. Influences of air pressure and nozzle diameter on print resolution

During the printing, the diameter of the printed straight filament (*d*) can be expressed as:

$$d = \frac{\alpha D}{\sqrt{V/C}}$$

where α is the die-swelling ratio, *D* is the nozzle diameter, *V* is the printing speed, and *C* is the extrusion speed.

According to previous reports,[1-2] air pressure *P* or nozzle diameter *D* both influence die-swelling ratio α and extrusion speed *C*, which further influence the diameter of printed filament *d*. Specifically, the increase in air pressure *P* would result in the increase in both die-swelling ratio α and extrusion speed *C*. So, at the same printing speed *V*, the filament diameter *d* is larger at a higher air pressure *P*. As the nozzle diameter *D* increases, the extrusion speed *C* would increase during the die-swelling ratio α decrease. Under the combined effect of *D*, *C*, and α, in the same printing speed *V*, the filament diameter *d* increases with the increased nozzle diameter *D*.

Although α and *C* cannot be quantitatively controlled, in the DIW printing technology, the printing speed *V* is the main controllable parameter because the nozzle diameter *D* is fixed and

there is a hysteresis in the variation of the air pressure P. Therefore, according to the volume conservation, the relationship can be transformed to

$$d_2 = d_1 \sqrt{\frac{V_1}{V_2}}$$

Where d_1 is the existing filament diameter, d_2 is the intended filament diameter, V_1 is the initial printing speed, and V_2 is the printing speed after the change.

Table S1. Printing parameters for each ink.

Recipes	Pressure (MPa)	Needle diameter (mm)	Printing speed (mm s^{-1})
InK 1-4-0.5	0.30	0.26	5
InK 1-4-1.0	0.18	0.26	4
InK 1-4-1.5	0.12	0.26	4.5

Movie S1. The 3D printing process.

Movie S2. Underwater capturing with the synthetic spiderweb.

Reference:

[1] L.-y. Zhou, J.-z. Fu, Q. Gao, P. Zhao, Y. He, *Adv. Funct. Mater.* **2019**, *30*, 1906683.

[2] L.-y. Zhou, Q. Gao, J.-z. Fu, Q.-y. Chen, J.-p. Zhu, Y. Sun, Y. He, *ACS Appl. Mater. Interfaces* **2019**, *11*, 23573.
